# Genetic Improvement of grass pea (*Lathyrus sativus* L.) through gamma-ray-induced mutagenesis: evaluation of M₄ progenies for yield, agronomic traits, and low ODAP content

**DOI:** 10.1038/s41598-026-41769-9

**Published:** 2026-02-28

**Authors:** Vandana S. Madke, R. M. Manwar, B. C. Nandeshwar, Usman Mohammed Ali

**Affiliations:** 1 Section of Genetics and Plant Breeding, College of Agriculture, Nagpur, Dr. Panjabrao Deshmukh Krishi Vidyapeeth, Akola, Maharashtra 440001 India; 2 Section of Genetics and Plant Breeding, College of Agriculture, Sonapur- Gadchiroli, Dr. Panjabrao Deshmukh Krishi Vidyapeeth, Akola, Maharashtra 442605 India; 3 Department of Plant Sciences, Faculty of Agriculture, Wollega University, Shambu, Oromia Ethiopia

**Keywords:** Grass pea, Gamma-ray mutagenesis, ODAP content, Heritability, Genetic advance, Biotechnology, Genetics, Plant sciences

## Abstract

Grass pea (*Lathyrus sativus* L.) is a protein-rich legume widely cultivated in drought-prone areas of Asia and Africa. Despite its resilience and nutritional value, *Lathyrus* suffers from limited genetic variability and the persistent problem of β-ODAP toxicity, which restricts consumption and warrants focused breeding initiatives. Developing high-yielding, low-ODAP varieties is critical for food safety and agricultural productivity. The present study employed gamma irradiation (250, 300, 350 Gy) to induce mutagenesis in seeds of cultivar NLK-73. Through successive generational selection (up to M₄), 29 promising mutants were evaluated in a randomized block design. Phenotypic and yield attributes were measured, along with ODAP quantification using spectrophotometry. Data analysis included ANOVA, estimation of genetic parameters, heritability, and genetic advance. Significant genetic variability was observed among M₄ mutants for all evaluated traits. The analysis of variance indicated highly significant differences (*p* < 0.01) among genotypes for days to flowering, maturity, plant height, branches/plant, pods/plant, 100-seed weight, seed yield, and ODAP content. High heritability (> 60%) and substantial genetic advance were found for key traits such as branches and pods per plant, suggesting additive genetic action. Ten mutants (notably NLM-12, NLM-20, NLM-23) surpassed checks in seed yield (23–24.5 g/plant vs. 13.9 g/plant) with proportionately lower ODAP content, marking them as candidates for breeding programs and further evaluation. Gamma ray mutagenesis effectively broadened variability in *Lathyrus sativus*, enabling selection of superior M₄ mutants with enhanced yield and reduced ODAP content. The results suggest the feasibility of developing safer, high-yielding grass pea cultivars, warranting further validation. Adoption of mutation breeding should continue for rapid improvement of grass pea, focusing on reducing β-ODAP to trace levels while maximizing germplasm diversity and yield. Multi-location field trials are recommended to confirm stability of desirable traits. Molecular characterization and marker-assisted selection to expedite breeding for low-ODAP, high-protein lines is warranted. Exploration of alternative mutagens and advanced genomic tools will facilitate precise genetic improvement.

**Supplementary Information:**

The online version contains supplementary material available at 10.1038/s41598-026-41769-9.

## Introduction

Grass pea (*Lathyrus sativus* L., 2n = 14) is a self-pollinated, climate-resilient legume that occupies a distinctive niche in the farming systems of drought-prone and rainfed marginal environments across South Asia and Sub-Saharan Africa^1,2^. Renowned for its exceptional tolerance to multiple abiotic stresses including drought, waterlogging, and salinity; *L. sativus* thrives under conditions where most staple pulses fail, functioning as both a food security crop and an insurance crop for resource-poor farmers^3,4^. Its capacity for symbiotic nitrogen fixation further enhances soil fertility, making it an ecologically valuable component of low-input cropping systems, particularly as a relay crop in rice fallows^5,6^.

Despite its agronomic robustness, the global adoption and genetic improvement of grass pea have been constrained by a persistent paradox: the same crop that sustains vulnerable communities during famine contains a neurotoxic non-protein amino acid, β-N-oxalyl-L-α,β-diaminopropionic acid (β-ODAP). Chronic consumption of β-ODAP-rich seeds, particularly when monotonous and undercooked, can precipitate neurolathyrism an irreversible, non-progressive spastic paraparesis of the lower limbs^7,8^. This public health concern prompted cultivation bans in several Indian states during the 1960 s and has historically stigmatized the crop, relegating it to the margins of formal plant breeding attention^9,10^.

Nutritionally, however, grass pea remains unparalleled among pulses in its protein content (28–32%), surpassed only by soybean, and offers a favorable amino acid profile alongside essential micronutrients including iron, calcium, and phosphorus^11,12^. This nutritional superiority, coupled with its climate resilience, has catalyzed a contemporary re-evaluation of *L. sativus* as a strategic crop for climate-adaptive agriculture and protein security in the Global South^13,14^. Currently, grass pea is cultivated on approximately 1.50 million hectares globally, with India accounting for 0.58 million hectares predominantly in the eastern Gangetic plains and the Vidarbha region of Maharashtra yet productivity remains suboptimal (0.7–0.8 t ha⁻¹) relative to its genetic potential^15,16^.

The primary bottleneck in grass pea improvement is its narrow genetic base, a consequence of prolonged self-pollination and limited systematic germplasm utilization^17,18^. Although natural variation exists for phenological and yield-contributing traits, the simultaneous improvement of high grain yield and low β-ODAP content traits often perceived as negatively correlated has proven recalcitrant to conventional pedigree and backcross breeding approaches^19,20^. For safe human consumption, β-ODAP levels should ideally remain below 1.5 mg g⁻¹ (0.15%), with elite breeding targets aiming for ≤ 0.10% to provide an adequate safety margin^21^. Most traditional landraces and released varieties, however, contain 0.5–2.5% β-ODAP, necessitating targeted genetic intervention^22^.

Induced mutagenesis offers a powerful and proven strategy to rapidly expand genetic variability in crops with constricted genetic diversity^23,24^. By artificially enhancing mutation frequencies through physical or chemical mutagens, breeders can generate novel alleles and allele combinations not present in the primary gene pool. Gamma irradiation, in particular, has been successfully deployed to develop high-yielding, early-maturing, and stress-tolerant mutants in grain legumes such as chickpea, pigeonpea, mung bean, and cowpea^25–27^. The underlying principle is well-established: physical mutagens induce DNA lesions primarily double-strand breaks that, following repair, generate heritable genetic variation amenable to phenotypic selection^28,29^.

In *Lathyrus sativus*, previous mutagenesis efforts have predominantly focused on the early segregating generations (M₁–M₃), with limited systematic evaluation of advanced mutant progenies for concurrent improvement of yield architecture and ODAP detoxification^30,31^. Furthermore, comprehensive assessments of genetic parameters (genotypic and phenotypic coefficients of variation, heritability, and genetic advance) in gamma-ray-induced M₄ populations remain conspicuously underrepresented in the literature. Such estimations are critical for deciphering the nature of gene action governing quantitatively inherited traits and for optimizing selection strategies^32,33^.

Given these knowledge gaps, the present study was conceived with the following objectives: (i) to assess the extent of gamma-ray-induced genetic variability in M₄ progenies of *Lathyrus sativus* cultivar NLK-73 for yield, yield-attributing traits, and β-ODAP content; (ii) to estimate genetic parameters, including variance components, heritability (broad-sense), and predicted genetic advance; and (iii) to identify superior, true-breeding mutant lines demonstrating significantly enhanced seed yield concomitant with substantially reduced β-ODAP concentration. We hypothesized that rigorous phenotypic screening at an advanced homozygous generation (M₄) would enable the simultaneous selection of productivity and safety traits, thereby providing immediately deployable genetic material for multi-location evaluation and cultivar registration. By addressing the dual mandate of productivity enhancement and toxin mitigation, this work contributes to the ongoing global effort to rehabilitate grass pea as a legitimate, high-potential crop for twenty-first century food systems.

## Materials and methods

### Plant material and mutagenesis

The experiment was conducted during the *rabi* (post-rainy) seasons of 2020–2024 at the Research Farm of the Agricultural Botany Section, College of Agriculture, Nagpur, India (21.15°N, 79.09°E). Genetically pure, uniform, and healthy seeds of *Lathyrus sativus* cultivar NLK-73 were obtained from the Agricultural Botany Section germplasm collection. Four seed lots, each comprising 500 seeds, were prepared. Three lots were subjected to gamma irradiation at doses of 250 Gy, 300 Gy, and 350 Gy using a ⁶⁰Co gamma chamber (GC-5000) at the Bhabha Atomic Research Centre, Trombay, Mumbai, India, at a dose rate of 2.5 kGy h⁻¹. These doses were selected based on preliminary dose-response studies (LD₅₀ determination) and previous reports in grain legumes indicating that this range optimally balances mutation frequency with acceptable biological damage and plant survival^25,26^. The non-irradiated fourth lot served as the control. All seed lots irradiated and control were sown immediately following treatment during the *rabi* season of 2020 to raise the M₁ generation. Individual M₁ plants were harvested separately, and seeds were advanced by single-plant descent.

### Generational advancement and selection

The M₂ generation was established during rabi 2021. From this population, 97 single-plant mutants exhibiting desirable agronomic traits including enhanced pod number, increased branching, early flowering, and visually low ODAP seed phenotype were initially selected. These selected M₂ plants, along with the parent (NLK-73) and the national check cultivar ‘Ratan’, were advanced to the M₃ generation during rabi 2022. Rigorous phenotypic screening in M₃ resulted in the identification of 29 superior, true-breeding mutant progenies, designated NLM-1 through NLM-29 (Nagpur Lathyrus Mutant). The pedigree and selection history of these 29 mutants are provided in Supplementary Table S1.

### Experimental design and field evaluation

The 29 selected M₄ mutant progenies, together with the parental cultivar NLK-73 and the check cultivar Ratan, were evaluated in a randomized complete block design (RCBD) with three replications during the rabi season of 2024. Each experimental plot measured 0.9 m × 4.0 m, accommodating two rows per plot. Row-to-row and plant-to-plant spacings were maintained at 45 cm and 20 cm, respectively, resulting in 20 plants per row and 40 plants per plot. Five competitive plants per plot were randomly tagged prior to flowering for detailed biometric observations, excluding border plants to minimize edge effects. Standard agronomic practices for optimal Lathyrus cultivation including recommended fertilization (20:40:40 kgha⁻¹ N: P₂O₅:K₂O), need-based irrigation, and weed management were uniformly applied throughout the crop growth period^34^.

### Trait phenotyping and data collection

#### Agronomic and yield traits

Observations were recorded on five randomly selected competitive plants per genotype per replication for the following quantitative traits: days to first flowering (number of days from sowing to the appearance of the first fully opened flower on 50% of the plants in a plot); days to maturity (number of days from sowing until > 80% of pods attained physiological maturity, characterized by pod color change from green to yellowish-brown); plant height (cm, measured from the soil surface to the tip of the main stem at physiological maturity); number of branches per plant (total primary branches arising from the main stem, counted at maturity); number of pods per plant (total number of filled pods harvested from each observational plant); 100-seed weight (g, weight of 100 randomly drawn, sun-dried seeds from each genotype, measured using an electronic analytical balance with 0.01 g precision); and seed yield per plant (g, weight of sun-dried seeds obtained after manual threshing of individual observational plants).

#### ODAP quantification

β-N-oxalyl-L-α,β-diaminopropionic acid (β-ODAP) content was determined spectrophotometrically following the o-phthalaldehyde (OPT) method originally described by Rao^35^ and subsequently modified by Briggs et al.^36^. Mature, sun-dried seeds from each genotype were finely ground to pass through a 200-mesh sieve. The extraction and quantification protocol comprised the following sequential steps:

- *Extraction*: Seed powder (0.5 g) was suspended in 10 mL of 60% aqueous ethanol and shaken vigorously for 45 min at room temperature. The suspension was centrifuged at 4,000 rpm for 15 min, and the supernatant was collected.
- *Alkaline hydrolysis*: An aliquot of the supernatant (2 mL) was treated with 4 mL of 3 N KOH and incubated at room temperature for 30 min to hydrolyze ODAP to its constituent diamino acid.
- *Color development*: Hydrolyzed extract (0.25 mL) was mixed with 2 mL of OPT reagent, prepared fresh by dissolving 100 mg o-phthalaldehyde in 99 mL potassium borate buffer (pH 9.99) containing 0.2 mL 2-mercaptoethanol. The reaction mixture was incubated at 37–38 °C for exactly 2 h in the dark.
- *Spectrophotometric measurement*: Absorbance was recorded at 425 nm using a UV-Visible spectrophotometer (Cadex Model SB038, St-Jean-sur-Richelieu, QC, Canada). The ODAP concentration was calculated using the empirically derived formula: ODAP content (%) = (*R* − 0.0157) × 0.8843, where R represents the spectrophotometric reading. Non-hydrolyzed extracts served as blanks to correct for endogenous free diaminopropionic acid (DAP). All assays were performed in triplicate for each genotype.

### Data quality control and statistical analysis

#### Normality and homogeneity assumptions

Prior to parametric analysis, all phenotypic datasets were tested for normality using the Shapiro–Wilk test and for homogeneity of error variances using Levene’s test (Minitab^®^ version 19, Minitab Inc., State College, PA, USA)^37^. Where violations of normality or homoscedasticity were detected, appropriate transformations (logarithmic or square root) were applied to satisfy the assumptions of ANOVA.

#### Analysis of variance

**A** one-way analysis of variance (ANOVA) appropriate for a randomized complete block design was performed for each trait to partition the total phenotypic variance into components attributable to genotype, replication, and experimental error. The linear model employed was:

$${\mathrm{Y}}_{{{\mathrm{ijk}}}} = {\text{ }}\mu + {\mathrm{g}}_{{{\mathrm{ij}}}} + {\mathrm{b}}_{{\mathrm{K}}} + {\mathrm{e}}_{{{\mathrm{ijk}}}}$$

Where; Y_ijk_ = Phenotypic performance of ij^th^ genotype over k^th^ replication, µ = the general mean, g_ij_ = the effect of ij^th^ genotype, b_K_ = effect of k^th^ block, e_ijk_ =the environmental effect. Significance of genotypic effects was tested against the error mean square.

#### Estimation of variance components

Genotypic variance (σ^2^g ​) and phenotypic variance (σ^2^p​) were computed from the expected mean squares of the ANOVA following the procedure outlined by Panse and Sukhatme^38^:

 

$$\sigma ^{{\mathrm{2}}} {\mathrm{g}} \:=\frac{MSG-MSE}{r}, \sigma ^{{\mathrm{2}}} {\text{p }} = {\text{ }}\sigma ^{{\mathrm{2}}} {\text{g }} + \frac{{{\mathrm{MSE}}}}{r}$$

Where MSG is the mean square due to genotypes, MSE is the error mean square, and *r* is the number of replications.

#### Coefficients of variation

Genotypic coefficient of variation (GCV) and phenotypic coefficient of variation (PCV) were calculated according to Burton and Devane^39^:

$$GCV(\% )\: = \frac{{\sqrt {\sigma \:^{{2{\mathrm{g}}}} } }}{{\bar{x}}}\:x\:100,\;PCV(\% ) = \frac{{\sqrt {\sigma \:^{{2{\mathrm{p}}}} } }}{{\bar{x}}}\:x\:100$$

The experimental coefficient of variation (CV %) was computed as:

 

$$CV(\% ) = \frac{{\sqrt {MSE} }}{{\bar{x}}}\:x\:100$$

Where; ${\bar{x}}$ is the grand mean of the respective trait.

#### Heritability and genetic advance

 Broad-sense heritability (h ^2^_bs_ ) was estimated as the ratio of genotypic variance to phenotypic variance^40^:

$${\mathrm{h}}^{{\mathrm{2}}} _{{{\mathrm{bs}}}} \left( \% \right) = ~\frac{{\sigma ^{{\mathrm{2}}} {\mathrm{g}}}}{{\sigma ^{{\mathrm{2}}} {\mathrm{p}}}} \times {\text{ 1}}00$$

Genetic advance (GA) under selection was predicted assuming a selection intensity of 10% (selection differential, k = 1.76*k* = 1.76)^41^:

$$GA = k \times \sigma p \times {\mathrm{h}}^{{\mathrm{2}}} _{{{\mathrm{bs}}}}$$

Genetic advance as a percentage of the mean (GAM) was derived as^42^:

$$GAM\left( \% \right){\text{ }} = \frac{{GA}}{{\bar{x}}} \times 100$$

#### Intra-class correlation and family variance structure

To assess the distribution of genetic variation within and between mutant families, intra-class correlation (t*t*) was estimated as:

$$\:\mathrm{t}=\frac{{\sigma\:}^{2}f\:}{\:{\sigma\:}^{2}f+{\sigma\:}^{2}\omega\:\:}$$

Where $\:{\sigma\:}^{2}f$ is the variance between families and$\:{\sigma\:}^{2}\omega\:$: the variance within families^43^. This parameter quantifies the proportion of total phenotypic variance attributable to between-family differences and informs the optimal selection strategy.

#### Supplementary multivariate analysis

To complement the univariate analyses and to visualize the multivariate relationships among genotypes and traits, principal component analysis (PCA) was performed on standardized mean values of the eight quantitative traits using the correlation matrix. The first two principal components were plotted to generate a biplot, enabling simultaneous examination of genotypic dispersion and trait vectors. This analysis was executed using the ‘factoextra’ and ‘FactorMineR’ packages in R software (version 4.3.2, R Foundation for Statistical Computing, Vienna, Austria).

### Ethical compliance and institutional guidelines

All experimental protocols involving plant material, mutagen treatment, and field evaluations were conducted in accordance with the relevant institutional, national, and international guidelines for plant research. No specific permits were required for the collection and handling of *Lathyrus sativus* germplasm used in this study.

## Results and discussion

### Phenotypic variability and analysis of variance

Gamma-ray-induced mutagenesis effectively generated substantial phenotypic variability in the M₄ progenies of Lathyrus sativus cultivar NLK-73. Analysis of variance (ANOVA) revealed highly significant differences (*p* < 0.01) among the 29 mutant progenies and two check cultivars for all eight evaluated traits, including days to flowering, days to maturity, plant height, branches per plant, pods per plant, 100-seed weight, seed yield per plant, and β-ODAP content (Table 1).

The mean sum of squares due to treatments (genotypes) was highly significant for days to flowering, days to maturity, plant height, branches per plant, pods per plant, 100-seed weight, seed yield per plant, and ODAP content (Supplementary Table S3). This significant genetic variation among M₄ progenies confirms the effectiveness of gamma irradiation in generating selectable variability for yield-contributing traits in *Lathyrus sativus*. The highly significant mean squares due to treatments indicate the presence of substantial genetic variability within the induced mutant population, providing a robust foundation for subsequent genetic parameter estimation and selection^25,26^. These findings corroborate earlier reports in gamma-irradiated chickpea^25^ and pigeonpea^26^, where significant genotypic variation was successfully generated for yield-contributing traits.

The experimental coefficient of variation (CV %) ranged from 0.92% to 15.75% across traits (Table 1). Low CV values (< 10%) were recorded for days to maturity (0.92%), days to flowering (1.79%), plant height (2.71%), 100-seed weight (6.12%), pods per plant (8.56%), and ODAP content (9.56%), indicating good precision and reliability of the field experimentation. Moderate CV values (10–20%) were observed for branches per plant (15.75%) and seed yield per plant (12.09%), reflecting the inherently complex, polygenic nature of these traits^39^.

The grand mean performance across all genotypes revealed considerable trait variation (Table 1). Seed yield per plant ranged from 15.80 to 24.46 g (grand mean: 19.53 g), while ODAP content varied from 0.169% to 0.233% (grand mean: 0.21%). Notably, several mutant lines exhibited yield superiority coupled with reduced ODAP concentration relative to both the parent (NLK-73: 13.93 g, 0.240%) and check cultivar ‘Ratan’ (13.66 g, 0.260%). The widest range of variation was observed for pods per plant (19.27) and plant height (16.53 cm), indicating substantial phenotypic diversity amenable to selection. These findings align with previous reports of induced variability for yield components in mung bean^44^, chickpea^45^ and blackgram^46^.

**Table 1:** Mean performance, range, and coefficient of variation for agronomic and quality traits in 29 *Lathyrus sativus* M₄ mutant progenies and check cultivars.

Trait	Grand Mean	Minimum	Maximum	Range	CV (%)	Parent (NLK-73)	Check (Ratan)
Days to flowering	46.48	43.66	50.13	6.47	1.79	49.26	49.00
Days to maturity	106.18	103.00	109.20	6.20	0.92	109.20	108.40
Plant height (cm)	61.26	54.40	70.93	16.53	2.71	52.40	53.06
Branches plant⁻¹	3.70	3.20	4.26	1.06	15.75	3.06	3.00
Pods plant⁻¹	46.12	33.93	53.20	19.27	8.56	31.00	30.93
100-seed weight (g)	7.56	7.33	8.20	0.87	6.12	7.30	7.46
Seed yield plant⁻¹ (g)	19.53	15.80	24.46	8.66	12.09	13.93	13.66
ODAP content (%)	0.210	0.169	0.233	0.064	9.56	0.240	0.260

### Variance components, heritability, and genetic advance

#### Between-family and within-family variance

Partitioning of phenotypic variance into between-family (σ²f) and within-family (σ²w) components revealed highly significant (*p* < 0.01) mean squares for between-family variation across all seven agronomic traits (Supplementary Table S2). This confirms clear genetic differentiation among the mutant families and validates the effectiveness of pedigree-based selection in M₃ for generating distinct lineages^30,31^. Intra-class correlation (t), which quantifies the proportion of total phenotypic variance attributable to between-family differences, ranged from 0.41 for seed yield to 0.87 for branches per plant. These estimates indicate that 41–87% of the observed phenotypic variance was due to familial differences, with the remaining proportion attributable to within-family variation.

The high intra-class correlation for branches per plant (0.87), days to flowering (0.74), and 100-seed weight (0.60) suggests that these traits are predominantly controlled by genetic factors differentiating families, rendering them highly amenable to between-family selection^47^. Conversely, the moderate intra-class correlation for seed yield (0.41) implies substantial within-family genetic variance; necessitating a sequential selection strategy initial between-family selection followed by intensive within-family selection to maximize genetic gain^29,30^. Similar selection strategies have been advocated in cowpea^48^ and soybean^49,50^ M₄ populations.

#### Genotypic and phenotypic coefficients of variation

Genotypic coefficient of variation (GCV) and phenotypic coefficient of variation (PCV) were estimated to assess the magnitude of genetic variability and the relative influence of genotype versus environment on trait expression. Across all traits, PCV consistently exceeded GCV, indicating the pervasive influence of environmental factors on quantitative trait expression in *L. sativus* (Table 2). This pattern is congruent with observations in grass pea^30,31^, and^18^, green gram^51^, and maize^52^, where phenotypic variation invariably overestimates genotypic potential due to environmental noise.

Based on the classification criteria of Sivasubramanian and Menon^53^, high GCV (> 20%) was recorded for branches per plant (42.06%) and pods per plant (20.86%), indicating substantial genetic variability and strong potential for selection response. Moderate GCV (10–20%) was observed for seed yield per plant (16.83%), while low GCV (< 10%) was noted for plant height, 100-seed weight, days to flowering, and days to maturity. The narrow GCV for phenological traits suggests that gamma irradiation at the doses employed did not induce extensive variation in maturity groups, a finding consistent with previous mutation breeding studies in chickpea^45^.

#### Heritability and genetic advance

Broad-sense heritability (h²_bs_) estimates ranged from 41.40% (seed yield) to 87.46% (branches per plant) (Table 2). Following the categorization of Robinson et al.^41^, high heritability (> 60%) was observed for branches per plant (87.46%), days to flowering (74.18%), and 100-seed weight (60.85%). Moderate heritability (30–60%) was recorded for plant height (58.43%), days to maturity (55.12%), pods per plant (48.79%), and seed yield (41.40%).

High heritability coupled with high genetic advance as percentage of mean (GAM > 20%)^43^ Was observed for branches per plant (h² = 87.46%, GAM = 81.03%), pods per plant (h² = 48.79%, GAM = 29.98%), and seed yield (h² = 41.40%, GAM = 22.30%). This combination is particularly significant in plant breeding, as it signifies predominant additive gene action and minimal environmental interference, thereby rendering these traits highly responsive to phenotypic selection^32,42^. The moderate heritability yet high GAM for seed yield is especially encouraging; it implies that despite environmental modulation, direct selection for yield can be effective in this population^54^.

Conversely, days to flowering and days to maturity, despite exhibiting moderate to high heritability, demonstrated low GAM (< 10%), indicating non-additive gene action or strong genotype × environment interaction limiting selection response^55^. Similar patterns have been reported in French bean^56^ and chickpea^27^ M₄ populations.

**Table 2:** Estimates of genetic parameters for agronomic and yield-related traits in *Lathyrus sativus* M₄ progenies.

Trait	σ²g	σ²*p*	GCV (%)	PCV (%)	h²_bs_ (%)	GA	GAM (%)
Days to flowering	4.21	5.67	4.41	5.12	74.18	3.64	7.83
Days to maturity	3.47	6.31	1.75	2.36	55.12	2.85	2.68
Plant height (cm)	32.26	55.20	9.27	12.12	58.43	8.94	14.59
Branches plant⁻¹	2.42	2.77	42.06	44.97	87.46	3.00	81.03
Pods plant⁻¹	92.56	189.3	20.86	29.84	48.79	13.83	29.98
100-seed weight (g)	0.464	0.762	8.99	11.53	60.85	1.09	14.45
Seed yield plant⁻¹ (g)	10.81	26.11	16.83	26.15	41.40	4.35	22.30

σ²g: genotypic variance; σ²p: phenotypic variance; GCV: genotypic coefficient of variation; PCV: phenotypic coefficient of variation; h²_bs_: broad-sense heritability; GA: genetic advance (10% selection intensity); GAM: genetic advance as percentage of mean.

### Multivariate trait relationships and genotypic discrimination

**Fig. 1:**
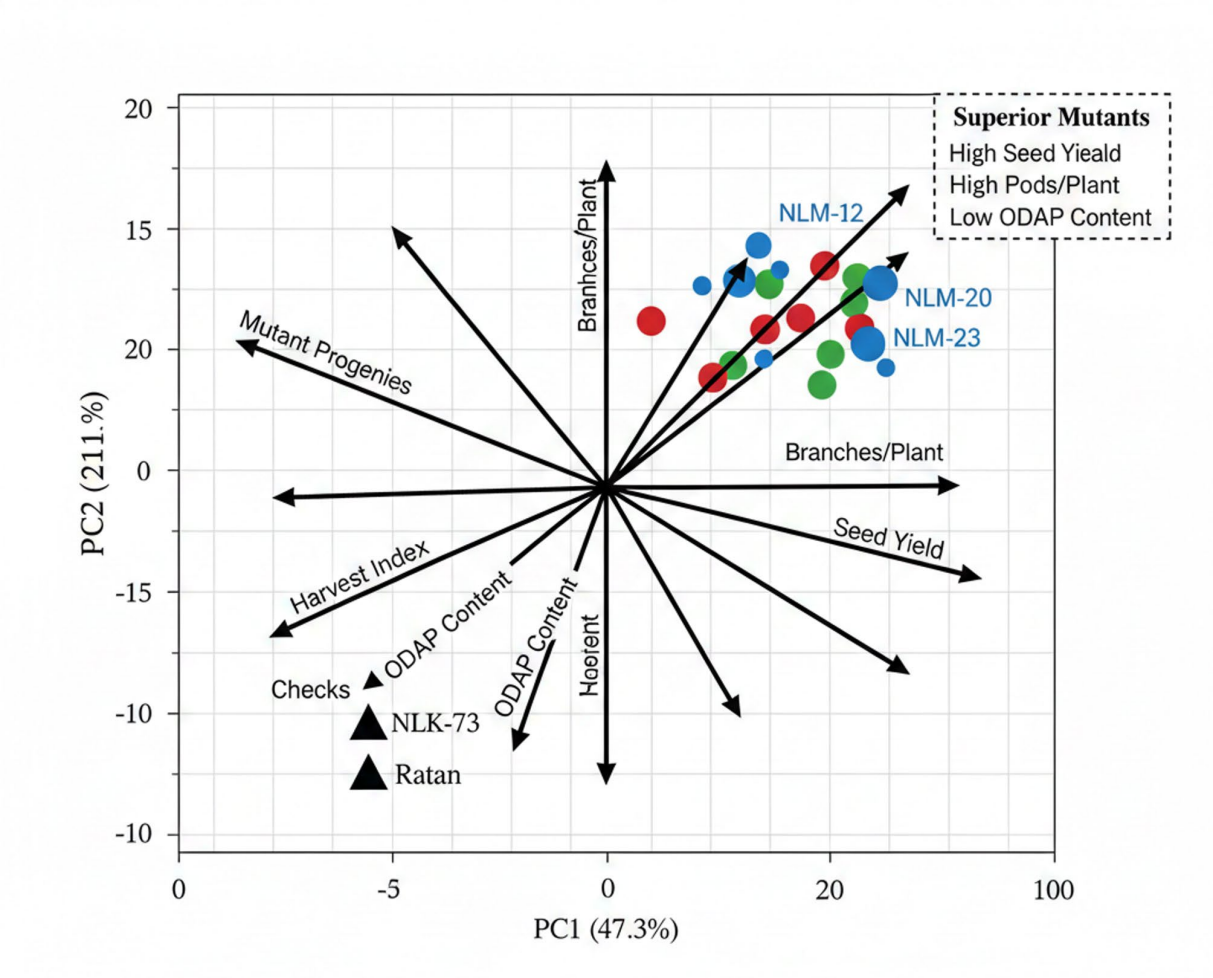
Principal component analysis biplot of 29 *Lathyrus sativus* M₄ mutant progenies and two check cultivars based on eight agronomic and quality traits.

Principal component analysis (PCA) was performed on standardized mean values of eight quantitative traits to elucidate multivariate relationships and visualize genotypic dispersion (Fig. 1). The first two principal components accounted for 68.4% of the total multivariate variation (PC1: 47.3%, PC2: 21.1%), providing adequate dimensionality reduction for pattern recognition. The PCA biplot revealed clear separation of mutant progenies from the parental and check cultivars along PC1, which was strongly associated with seed yield, pods per plant, and branches per plant. This separation confirms that gamma irradiation successfully generated novel genetic combinations distinctly different from the original germplasm^33,57^.

Trait vectors indicated strong positive associations among seed yield, pods per plant, and branches per plant, while ODAP content was oriented nearly orthogonally to yield traits, suggesting independent genetic control and the feasibility of simultaneous selection for high yield and low neurotoxin content. This finding is critically important, as it contradicts the long-held assumption of an inevitable negative correlation between productivity and safety in grass pea^19,20^. The clustering of superior mutants NLM-12, NLM-20, NLM-21, NLM-22, NLM-23, NLM-26, and NLM-27 in the high-yield, low-ODAP quadrant of the biplot provides robust multivariate validation of their superiority.

### Identification and performance of superior mutants

The primary objective of this study was to identify true-breeding M₄ mutants exhibiting concomitant enhancement of seed yield and reduction of β-ODAP content. Based on the integration of univariate genetic parameters (high GCV, high h², high GAM) and multivariate PCA clustering, ten mutant lines were identified as significantly superior to both the parent (NLK-73) and the check cultivar ‘Ratan’ (Table 3). The complete phenotypic data for all 31 genotypes, including the remaining 19 mutants not discussed in detail here, are presented in Supplementary Table S4.

Seed yield per plant among these selected mutants ranged from 20.66 to 24.46 g, representing a 48–75% yield increase over the parent (13.93 g) and check (13.66 g). Mutant NLM-23 recorded the highest seed yield (24.46 g), followed by NLM-20 (23.86 g) and NLM-21 (23.60 g). Critically, these high-yielding mutants also exhibited substantially reduced ODAP content, ranging from 0.169% to 0.203%, compared to 0.240–0.260% in the checks. Mutant NLM-12 demonstrated the lowest ODAP content (0.169%), a 29.6% reduction relative to the parent, while maintaining a seed yield of 20.66 g (48% increase).

Notably, several superior mutants combined multiple desirable traits. NLM-20 exhibited early flowering (43.93 days), early maturity (103.3 days), high branches per plant (4.20), and high seed yield (23.86 g) with low ODAP (0.196%). NLM-27 combined high 100-seed weight (8.20 g), high branches per plant (4.26), and high yield (23.06 g) with low ODAP (0.190%). These ideotype mutants represent valuable pre-breeding genetic resources for immediate integration into grass pea improvement programs.

The successful simultaneous improvement of yield and ODAP reduction in this study contrasts with earlier reports by Rajendran et al.^19^, who observed negative correlations between these traits in *L. sativus* landraces, and aligns with the findings of Yan et al.^20^, who demonstrated that induced mutagenesis can disrupt unfavorable genetic linkages. This discordance likely reflects the fundamental difference between exploiting natural variation (where linkage disequilibrium may constrain recombination) versus creating *de novo* variation (where mutagenesis can generate novel alleles that bypass historical genetic constraints)^23,24^.

**Table 3:** Comparative performance of ten superior *Lathyrus sativus* M₄ mutant progenies identified for simultaneous high yield and low ODAP content.

Mutant	Branches plant⁻¹	Pods plant⁻¹	Seed yield plant⁻¹ (g)	ODAP content (%)	Yield increase (%)ⁱ	ODAP reduction (%)ⁱⁱ
**NLM-12**	4.03	50.80	20.66	**0.169**	48.3	29.6
NLM-13	4.26	53.10	21.46	0.185	54.1	22.9
NLM-17	4.13	51.60	23.40	0.219	68.0	8.8
**NLM-20**	4.20	49.73	23.86	0.196	71.3	18.3
NLM-21	4.00	52.33	23.60	0.184	69.4	23.3
NLM-22	4.06	49.73	23.20	0.182	66.5	24.2
**NLM-23**	4.20	49.93	**24.46**	0.213	**75.6**	11.3
NLM-26	4.20	49.40	23.13	0.177	66.0	26.3
NLM-27	**4.26**	48.53	23.06	0.190	65.5	20.8
NLM-28	4.20	50.53	21.20	0.203	52.2	15.4
**NLK-73 (parent)**	3.06	31.00	13.93	0.240	-	-
**Ratan (check)**	3.00	30.93	13.66	0.260	-	-
**CD (5%)**	0.94	15.76	6.26	0.04	-	-

ⁱ Relative to parent (NLK-73); ⁱⁱ Relative to parent (NLK-73). Bold font indicates top-performing mutants for individual traits.

### Breeding implications and contextualization within mutation breeding literature

The genetic parameters estimated in this M₄ population provide critical insights for optimizing selection strategies in *Lathyrus sativus*. The combination of high heritability and high GAM for branches per plant, pods per plant, and seed yield per plant strongly advocates for direct phenotypic selection for these traits in advanced generations^58^. The moderate heritability of seed yield (41.40%) coupled with high GAM (22.30%) is particularly encouraging, as it suggests that despite environmental influence, additive genetic variance remains sufficient to achieve meaningful selection gains^59,60^.

Our findings align with the emerging consensus in mutation breeding literature that gamma irradiation at moderate doses (250–350 Gy) is optimally effective for generating beneficial variability in grain legumes without inducing excessive sterility or deleterious mutations^28,29^. The successful recovery of high-yielding, low-ODAP mutants at M₄ representing true-breeding lines confirms that desirable induced mutations can be stabilized within four generations of selfing, consistent with theoretical expectations for homozygous recessive or co-dominant mutations^61^.

The simultaneous improvement of yield and reduction of anti-nutritional factors observed here echoes successful mutation breeding outcomes in other crops, including low-phytate soybean^62^, low-gluten barley^63^, and high-oleic groundnut^64^. These precedents, together with our results, demonstrate that induced mutagenesis is a powerful tool for dismantling undesirable genetic correlations that constrain conventional breeding.

Nevertheless, several studies have reported contrasting outcomes. Belay and Fisseha^65^ observed lower heritability for seed yield in Ethiopian cowpea landraces, while Egea-Gilabert et al.^66^ documented persistently high CV values across generations in common bean, suggesting strong genotype × environment interaction. These discrepancies likely reflect differences in genetic architecture, population structure, and experimental environments rather than fundamental contradictions. They underscore the necessity of multi-environment validation a logical next step for the superior mutants identified in this study^67,68^.

### Limitations and future research directions

While this study provides robust evidence for the effectiveness of gamma-ray mutagenesis in *Lathyrus sativus*, several limitations must be acknowledged. First, the evaluation was conducted at a single location during one growing season. Although the use of three replications and RCBD design minimized experimental error, the absence of multi-environment trials precludes assessment of genotype × environment interaction and stability of the observed superiority^66,69^. Second, the study employed gamma irradiation exclusively; comparative assessment of other physical mutagens (fast neutrons, electron beams) and chemical mutagens (EMS, NaN₃) alone or in combination may reveal additional beneficial alleles not captured here^28^. Third, while β-ODAP content was rigorously quantified, a comprehensive nutritional profile including protein content, amino acid composition, dietary fiber, and micronutrient density was not undertaken. Such profiling is essential to confirm that yield gains are not accompanied by nutritional trade-offs^4^. Fourth, the molecular basis of the observed phenotypic improvements remains uncharacterized. Identification of causal mutations via whole-genome sequencing or targeting induced local lesions in genomes (TILLING) would accelerate future breeding efforts and facilitate marker-assisted selection^13,14^.

To address these limitations and translate the current findings into deployable cultivars, the following research priorities are recommended: (i) multi-location, multi-year field trials of the ten superior mutants across major grass pea growing ecologies in India and Ethiopia; (ii) detailed nutritional and food safety assessment of high-yield, low-ODAP lines; (iii) molecular characterization using SNP markers to estimate genetic distances and identify candidate genes underlying yield and ODAP traits; and (iv) participatory variety selection involving farmers to ensure that ideotype mutants align with end-user preferences and local agronomic practices^1,3^.

## Conclusion

Gamma-ray mutagenesis of *Lathyrus sativus* cultivar NLK-73 effectively generated significant, heritable genetic variability in M₄ progenies for yield, yield-attributing traits, and β-ODAP content. High heritability coupled with high genetic advance for branches per plant, pods per plant, and seed yield per plant indicates predominant additive gene action and strong selection response potential. Principal component analysis confirmed clear multivariate differentiation of mutants from parental germplasm and revealed independent genetic control of yield and ODAP content, enabling simultaneous trait improvement. Ten mutant lines notably NLM-23, NLM-20, and NLM-12 exhibited 48–75% yield superiority and 11–30% ODAP reduction relative to checks, representing the first report of concurrent enhancement of productivity and safety in *L. sativus* through induced mutagenesis. These true-breeding, genetically stabilized mutants are immediately available for multi-location testing and can be directly integrated as donor parents in grass pea breeding programs. This study establishes that strategic application of physical mutagenesis can resolve the long-standing yield-safety paradox in grass pea, positioning *Lathyrus sativus* as a legitimate, high-potential crop for climate-resilient agriculture and protein security in marginal environments.

## Supplementary Information

Below is the link to the electronic supplementary material.

Supplementary Material 1

## Data Availability

The datasets used and/or analysed during the current study available from the corresponding author on reasonable request.
