# Supplementary material for: Genetic Improvement of grass pea (Lathyrus sativus L.) through gamma-ray-induced mutagenesis: evaluation of M₄ progenies for yield, agronomic traits, and low ODAP content
Source: Sci Rep. 2026 Feb 28;16:11453. doi: 10.1038/s41598-026-41769-9 (PMC13056989; doi:10.1038/s41598-026-41769-9)
Supplement: Supplementary file 1 — Supplementary Material 1 [file 41598_2026_41769_MOESM1_ESM.docx]

***SUPPLEMENTARY INFORMATION***

**Genetic Improvement of Grass pea (*Lathyrus sativus* L.) through Gamma-Ray-Induced Mutagenesis: Evaluation of M₄ Progenies for Yield, Agronomic Traits, and Low ODAP Content**

Vandana S Madke^1^, R.M Manwar^1^, B C Nandeshwar^2^, Usman Mohammed Ali*^3^

^1^Section of Genetics and Plant Breeding, College of Agriculture, Nagpur, PIN 440001,

Dr. Panjabrao Deshmukh Krishi Vidyapeeth, Akola, Maharashtra, India.

^2^Section of Genetics and Plant Breeding, College of Agriculture, Sonapur-Gadchiroli, PIN-442 605, Dr. Panjabrao Deshmukh Krishi Vidyapeeth, Akola, Maharashtra, India.

^3^Department of Plant Sciences, Faculty of Agriculture , Wollega University , Shambu , Oromia, Ethiopia. *Corresponding author: [ausmanmohammed77@gmail.com](mailto:ausmanmohammed77@gmail.com)

**Supplementary Table S1:** Pedigree, mutagen dose, and selection history of 29 Lathyrus sativus M₄ mutant progenies derived from gamma-irradiated cultivar NLK-73.

| **Sr. No.** | **Mutants** | **Pedigree** | **Character for which Identified** |
| --- | --- | --- | --- |
| 1 | NLM-1 | NGP20-300Gy-245-4 | High yield and more pods |
| 2 | NLM-2 | NGP20-300Gy- 307-10 | High yield and more pods |
| 3 | NLM-3 | NGP20-300Gy-311-10 | High yield and more pods |
| 4 | NLM-4 | NGP20-300Gy-245-4 | High yield and more pods |
| 5 | NLM-5 | NGP20-300Gy-278-12 | High yield and more pods |
| 6 | NLM-6 | NGP20-350Gy-25-12 | High yield, more pods and more branches |
| 7 | NLM-7 | NGP20-350Gy-341-7 | High yield and more pods |
| 8 | NLM-8 | NGP20-350Gy- 344-4 | High yield, more pods, branch |
| 9 | NLM-9 | NGP20-350Gy-344-4 | High yield and more pods |
| 10 | NLM-10 | NGP20-350Gy-6-1 | High yield and more pods, more branches |
| 11 | NLM-11 | NGP20-350Gy-22-3 | High yield and more pods |
| 12 | NLM-12 | NGP20-350Gy-3-6 | High yield and more pods |
| 13 | NLM-13 | NGP20-350Gy-3-19 | High yield and more pods, more branches |
| 14 | NLM-14 | NGP20-350Gy-3-19 | High yield and more pods |
| 15 | NLM-15 | NGP20-350Gy-25-3 | High yield, more pods, bold seed |
| 16 | NLM-16 | NGP20-350Gy-25-3 | High yield and more pods |
| 17 | NLM-17 | NGP20-350Gy-25-12 | High yield, more pods and more branches |
| 18 | NLM-18 | NGP20-350Gy-25-12 | High yield and more pods |
| 19 | NLM-19 | NGP20-350Gy-25-10 | High yield and more pods |
| 20 | NLM-20 | NGP20-350Gy-25-10 | High yield and more pods and early flower |
| 21 | NLM-21 | NGP20-350Gy-31-7 | High yield and more pods |
| 22 | NLM-22 | NGP20-350Gy-32-7 | High yield and more pods |
| 23 | NLM-23 | NGP20-350Gy-32-14 | High yield, more pods and branches |
| 24 | NLM-24 | NGP20-350Gy-32-14 | High yield and more pods |
| 25 | NLM-25 | NGP20-350Gy-351-16 | High yield and more pods |
| 26 | NLM-26 | NGP20-350Gy-351-14 | High yield and more pods |
| 27 | NLM-27 | NGP20-350Gy-351-14 | High yield and more pods |
| 28 | NLM-28 | NGP20-350Gy-351-5 | High yield , more pods and early flower |
| 29 | NLM-29 | NGP20-350Gy-351-11 | High yield and more pods |
| 30 | NLK-73 (ch) | - | - |
| 31 | Ratan (ch) | - | - |

**(Source:** Bhabha Atomic Research Centre, Trombey, Mumbai,India).

**Supplementary Table S2:** Analysis of variance for between-family and within-family components with intra-class correlation estimates for seven agronomic traits in M₄ progenies.

| **Mean sum of squares** | | | | | | | | |
| --- | --- | --- | --- | --- | --- | --- | --- | --- |
| **Source of variation** | **df** | **Day to first flower** | **Days to maturity** | **Plant height (cm)** | **Number of branches plant^-1^** | **Number of pods plant^-1^** | **100 seeds weight (g)** | **Seed yield plant^-1^** |
| Between families | 30 | 64.66** | 55.01** | 506.84** | 2.45** | 1485.2** | 0.48** | 177.49** |
| Within families | 434 | 1.47 | 2.83 | 22.95 | 0.35 | 96.80 | 0.31 | 15.30 |
| Intra class correlation(t) |  | 0.74 | 0.55 | 0.58 | 0.87 | 0.48 | 0.60 | 0.41 |

** Significant at 1% level

**Supplementary Table S3:** Analysis of variance (ANOVA) for eight agronomic and quality traits evaluated in 29 Lathyrus sativus M₄ mutant progenies and two check cultivars under randomized complete block design.

| **Mean sum of squares** | | | | | | | | | |
| --- | --- | --- | --- | --- | --- | --- | --- | --- | --- |
| **Source of variation** | **df** | **Days to first flower** | **Days to maturity** | **Plant height (cm)** | **Number of branches plant^-1^** | **Number of pods plant^-1^** | **100 seeds weight (g)** | **Seed yield plant^-1^ (g)** | **ODAP content (%)** |
| Replication | 2 | 1.93 | 2.59 | 39.62 | 0.11 | 83.83 | 0.17 | 8.63 | 0.000164 |
| Treatment | 30 | 6.65** | 11.10** | 148.96** | 0.18** | 92.74** | 0.88** | 40.63** | 0.02** |
| Error | 60 | 2.23 | 5.03 | 17.77 | 0.08 | 38.57 | 0.41 | 6.91 | 0.000261 |

**Key:** ** Significant at 1% level

**Supplementary Table S4:** Mean performance of 29 *Lathyrus sativus* M₄ mutant progenies, parental cultivar NLK-73, and check cultivar Ratan for eight agronomic and quality traits.

| **Sr. No.** | **Genotype** | **Days to first flower** | **Days to maturity** | **Plant height (cm)** | **Branches plant⁻¹** | **Pods plant⁻¹** | **100-seed weight (g)** | **Seed yield plant⁻¹ (g)** | **ODAP content (%)** |
| --- | --- | --- | --- | --- | --- | --- | --- | --- | --- |
| **1** | **NLM-1** | 44.00 | 106.2 | 55.86 | 3.46 | 49.53 | 7.53 | 20.66 | 0.226 |
| **2** | **NLM-2** | 45.93 | 105.2 | 60.13 | 3.26 | 53.20 | 7.33 | 22.13 | 0.231 |
| **3** | **NLM-3** | 47.80 | 103.6 | 67.40 | 3.26 | 44.53 | 7.46 | 18.60 | 0.233 |
| **4** | **NLM-4** | 43.66 | 107.7 | 54.60 | 3.53 | 43.26 | 7.53 | 18.06 | 0.228 |
| **5** | **NLM-5** | 48.86 | 103.8 | 58.80 | 3.33 | 51.73 | 7.33 | 16.00 | 0.225 |
| **6** | **NLM-6** | 45.46 | 104.3 | 63.80 | 3.20 | 52.33 | 7.46 | 23.06 | 0.208 |
| **7** | **NLM-7** | 44.20 | 108.2 | 55.60 | 4.06 | 43.20 | 7.46 | 15.80 | 0.210 |
| **8** | **NLM-8** | 47.46 | 104.4 | 57.66 | 3.26 | 52.73 | 7.53 | 16.20 | 0.221 |
| **9** | **NLM-9** | 46.00 | 107.2 | 64.13 | 3.26 | 45.20 | 7.46 | 17.93 | 0.208 |
| **10** | **NLM-10** | 44.00 | 108.2 | 56.40 | 3.80 | 53.06 | 7.53 | 19.70 | 0.185 |
| **11** | **NLM-11** | 48.06 | 104.9 | 59.60 | 3.33 | 47.13 | 7.66 | 17.80 | 0.219 |
| **12** | **NLM-12** | 44.13 | 107.9 | 64.13 | 4.03 | 50.80 | 7.46 | 20.66 | **0.169** |
| **13** | **NLM-13** | 46.26 | 109.2 | 57.06 | **4.26** | **53.10** | 7.66 | 21.46 | 0.185 |
| **14** | **NLM-14** | 44.06 | 106.0 | 65.86 | 4.00 | 50.20 | 7.53 | 18.33 | 0.204 |
| **15** | **NLM-15** | 48.26 | 104.2 | 69.26 | 4.06 | 51.00 | 7.60 | 19.66 | 0.219 |
| **16** | **NLM-16** | 44.13 | 104.7 | 55.53 | 3.40 | 38.86 | 7.46 | 16.26 | 0.227 |
| **17** | **NLM-17** | 46.86 | 108.3 | 64.80 | 4.13 | 51.60 | 7.60 | 23.40 | 0.219 |
| **18** | **NLM-18** | 50.00 | 105.3 | **70.93** | 3.40 | 43.26 | 7.46 | 16.60 | 0.226 |
| **19** | **NLM-19** | 47.00 | 107.9 | 54.40 | 3.33 | 46.86 | 7.60 | 21.20 | 0.205 |
| **20** | **NLM-20** | **43.93** | **103.3** | 65.46 | 4.20 | 49.73 | 7.60 | 23.86 | 0.196 |
| **21** | **NLM-21** | 47.06 | 106.2 | 70.66 | 4.00 | 52.33 | 7.66 | 23.60 | 0.184 |
| **22** | **NLM-22** | 45.13 | **103.0** | 55.86 | 4.06 | 49.73 | 7.46 | 23.20 | 0.182 |
| **23** | **NLM-23** | 47.86 | 107.2 | 65.40 | 4.20 | 49.93 | 7.60 | **24.46** | 0.213 |
| **24** | **NLM-24** | 50.13 | 109.0 | 70.46 | 4.13 | 33.93 | 7.46 | 16.40 | 0.209 |
| **25** | **NLM-25** | 46.46 | 105.0 | 63.40 | 3.73 | 51.53 | 7.66 | 20.06 | 0.213 |
| **26** | **NLM-26** | 49.80 | 106.7 | 55.53 | 4.20 | 49.40 | 7.66 | 23.13 | 0.177 |
| **27** | **NLM-27** | 44.80 | 103.8 | 70.93 | **4.26** | 48.53 | **8.20** | 23.06 | 0.190 |
| **28** | **NLM-28** | 44.06 | 106.8 | 56.60 | 4.20 | 50.53 | 7.80 | 21.20 | 0.203 |
| **29** | **NLM-29** | 47.40 | 104.9 | 61.46 | 3.26 | 45.40 | 8.00 | 16.46 | 0.220 |
| **30** | **NLK-73 (Parent)** | 49.26 | 109.2 | 52.40 | 3.06 | 31.00 | 7.30 | 13.93 | 0.240 |
| **31** | **Ratan (Check)** | 49.00 | 108.4 | 53.06 | 3.00 | 30.93 | 7.46 | 13.66 | 0.260 |
|  | **Grand Mean** | **46.48** | **106.18** | **61.26** | **3.70** | **46.12** | **7.56** | **19.53** | **0.210** |
|  | **SE(m) ±** | **0.70** | **0.97** | **2.77** | **0.34** | **5.69** | **0.32** | **2.26** | **0.01** |
|  | **CD (p ≤ 0.05)** | **1.97** | **2.74** | **7.82** | **0.94** | **15.76** | **0.90** | **6.26** | **0.04** |
|  | **CV (%)** | **1.79** | **0.92** | **2.71** | **15.75** | **8.56** | **6.12** | **12.09** | **9.56** |

**NLK-73 (Number of branches-3.06)**


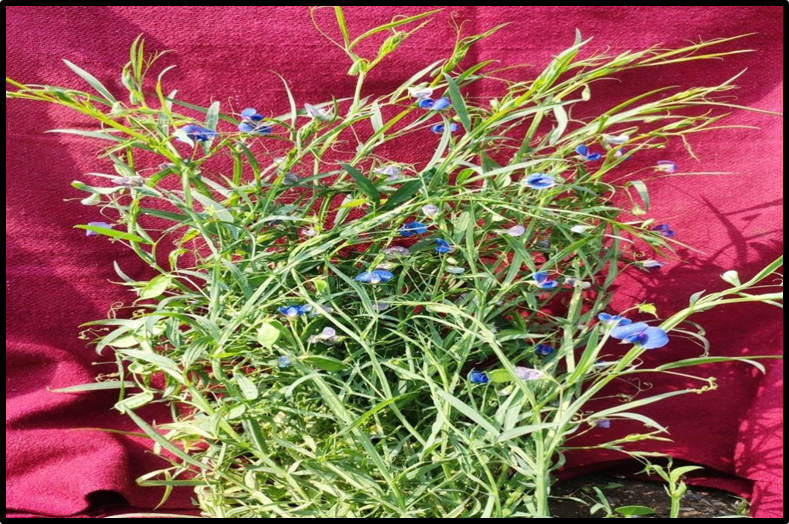


**NLK-73 (Number of pods-31)**

**
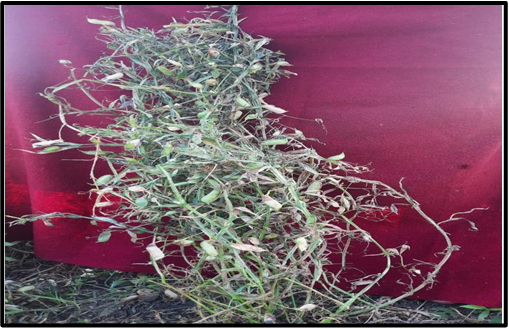
**

**NLM-13 and NLM-27 (Number of branches-4.20)**

**Plate 2:** Profuse branching mutant progeny along with check


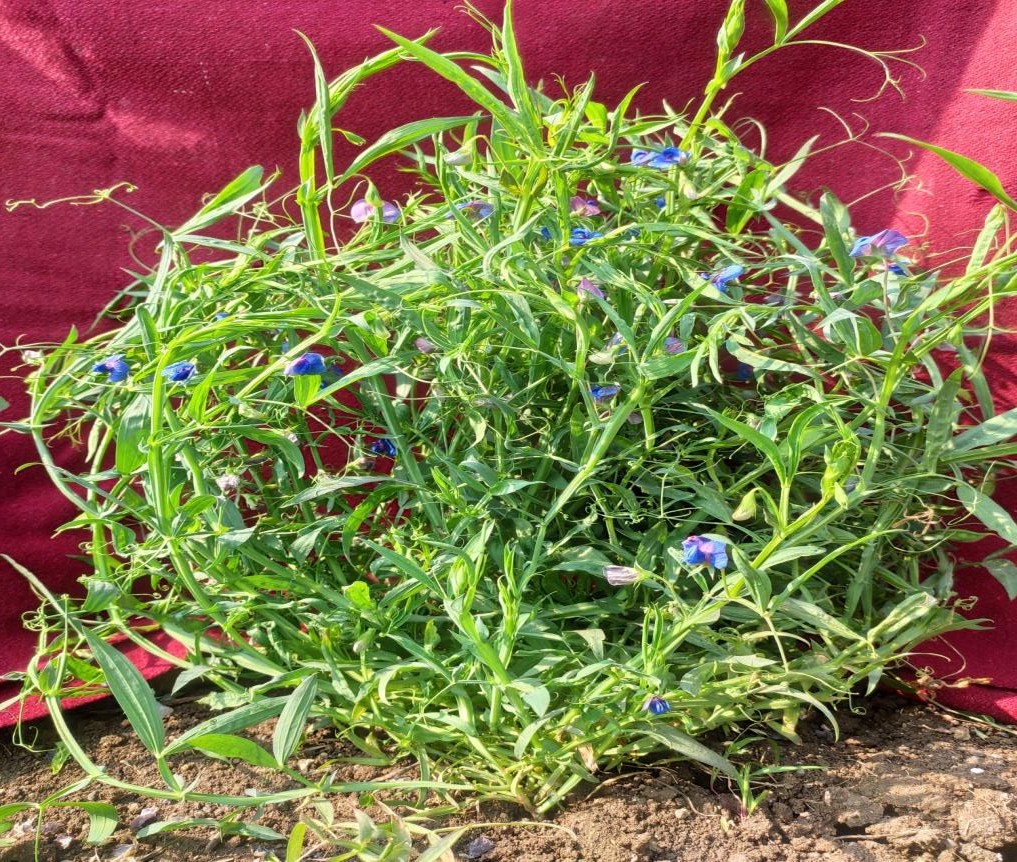

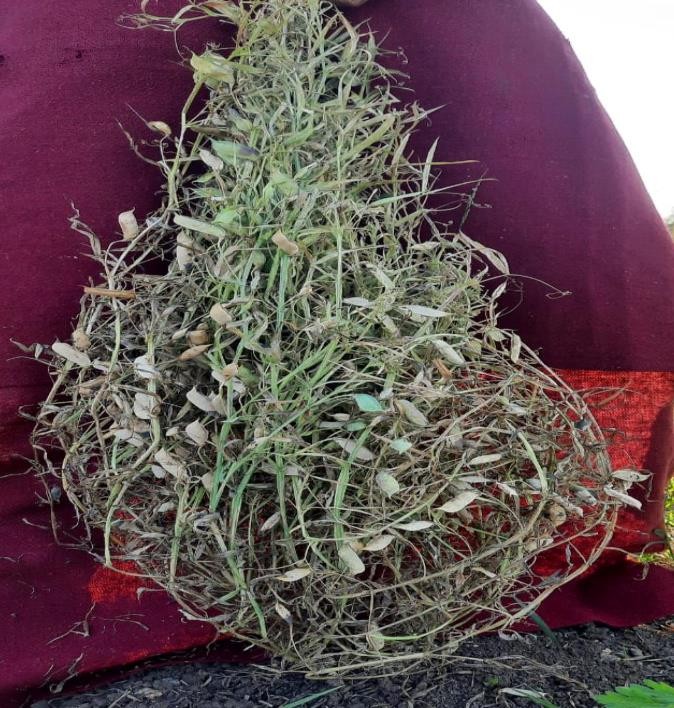


**NLK-73 (Number of pods-31)**

**Plate 3:** More number of pods mutant progeny along with check


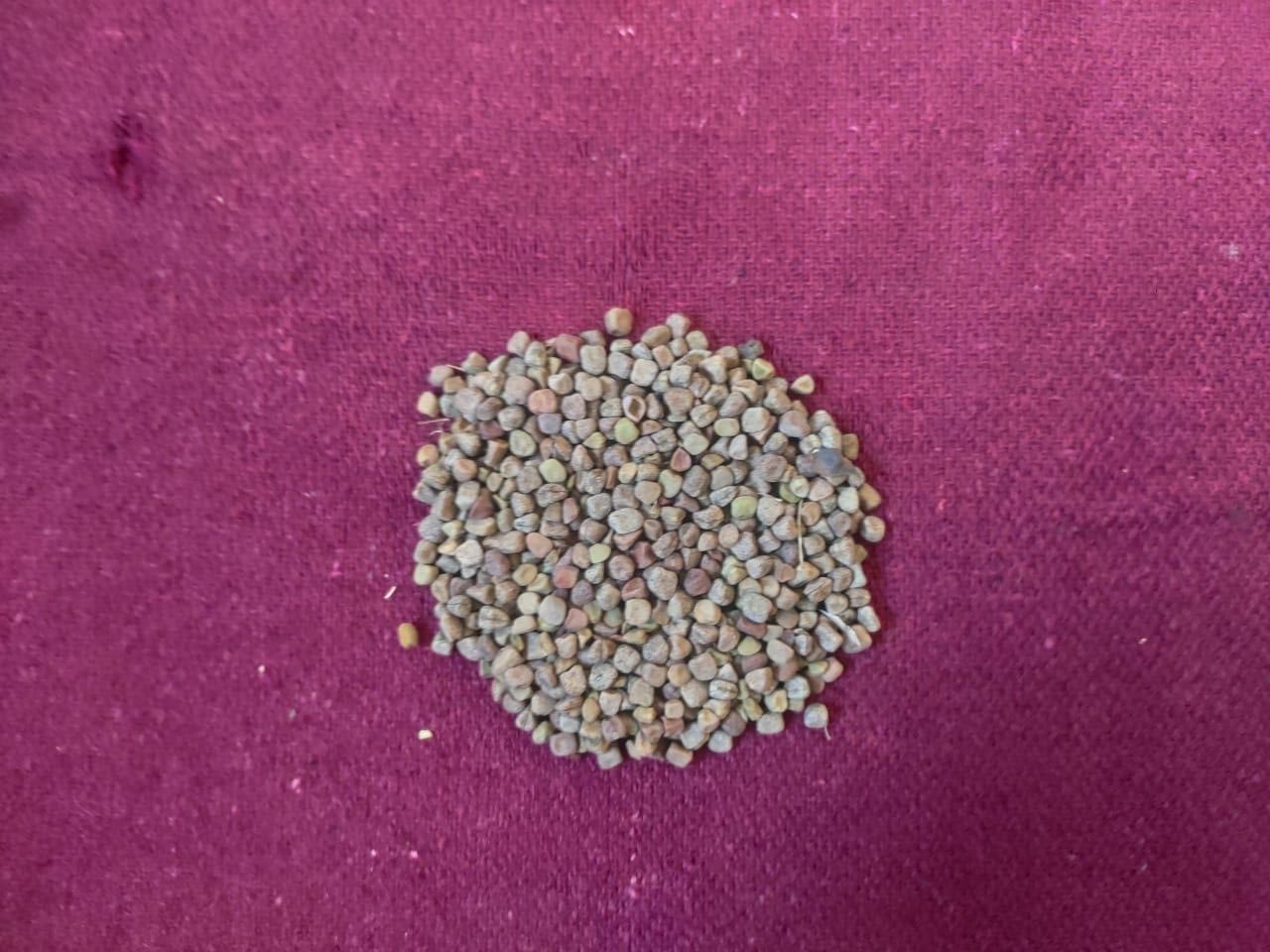


**NLM-23 (24.46g)**

**Plate 4:** High yield mutant along with check


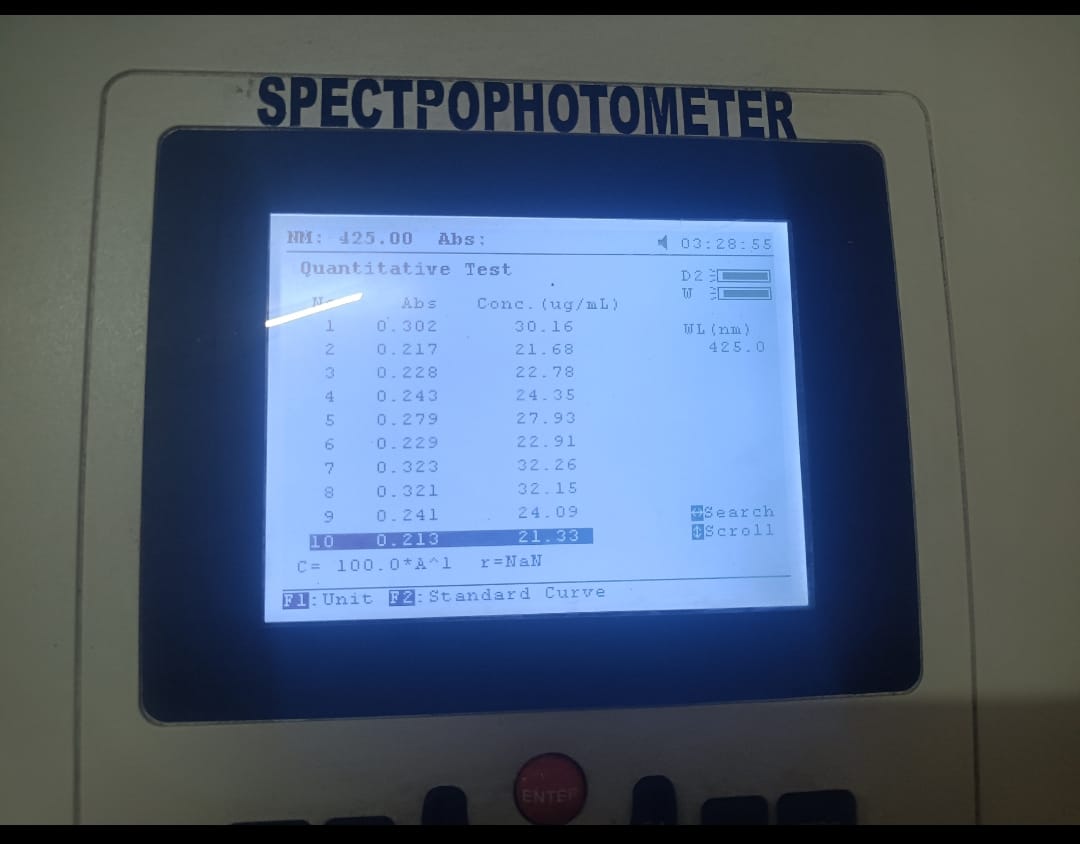


**Plate 5:** Laboratory analysis of Lathyrus genotypes for low ODAP content using spectrophotometer
